# Intravascular Imaging Guidance Reduce 1-Year MACE in Patients Undergoing Rotablator Atherectomy-Assisted Drug-Eluting Stent Implantation

**DOI:** 10.3389/fcvm.2021.768313

**Published:** 2021-10-27

**Authors:** Nattawut Wongpraparut, Paroj Bakoh, Kawin Anusonadisai, Namthip Wongsawangkit, Damras Tresukosol, Chunhakasem Chotinaiwattarakul, Rewat Phankingthongkum, Wiwun Tungsubutra, Narathip Chunhamaneewat, Korakoth Towashiraporn, Asa Phichaphop, Pariya Panchavinnin, Rungtiwa Pongakasira, Pradit Panchavinnin

**Affiliations:** ^1^Division of Cardiology, Department of Medicine, Faculty of Medicine Siriraj Hospital, Mahidol University, Bangkok, Thailand; ^2^Faculty of Medicine Siriraj Hospital, Her Majesty's Cardiac Center, Mahidol University, Bangkok, Thailand

**Keywords:** imaging guidance, heavy calcified lesion, rotablator atherectomy, drug-eluting stent implantation, 1-year MACE

## Abstract

**Objectives:** This study aimed to investigate the incidence of 1-year major adverse cardiac events (MACE) compared between intravascular imaging guidance and angiographic guidance in patients undergoing rotablator atherectomy (RA)-assisted percutaneous coronary intervention (PCI) with drug-eluting stent (DES) implantation.

**Methods:** This retrospective analysis included 265 consecutive patients with heavy calcified lesion who underwent RA-assisted PCI with DES implantation at our institution during the January 2016-December 2018 study period. This study was approved by the Siriraj Institutional Review Board. Patients were divided into either the angiographic guidance PCI group or the imaging guidance PCI group, which was defined as intravascular ultrasound or optical coherence tomography. The primary endpoint was 1-year MACE.

**Results:** Two hundred and sixty-five patients were enrolled, including 188 patients in the intravascular imaging guidance group, and 77 patients in the angiographic guidance group. One-year MACE was significantly lower in the imaging guidance group compared to the angiographic guidance group (4.3 *vs*. 28.9%, respectively; odds ratio (OR): 9.06, 95% CI: 3.82–21.52; *p* < 0.001). The 1-year rates of all-cause death (OR: 8.19, 95% CI: 2.15–31.18; *p* = 0.002), myocardial infarction (MI) (OR: 6.13, 95% CI: 2.05–18.3; *p* = 0.001), and target vessel revascularization (TVR) (OR: 3.67, 95% CI: 1.13–11.96; *p* = 0.031) were also significantly lower in the imaging guidance group compared with the angiographic guidance group. The rate of stroke was non-significantly different between groups.

**Conclusion:** In patients with heavy calcified lesion undergoing RA-assisted DES implantation, the intravascular imaging guidance significantly reduced the incidence of 1-year MACE, all-cause death, MI, and TVR compared to the angiographic guidance.

## Introduction

Superficial coronary calcified atherosclerotic plaque is a predictor of worse prognosis (1) after percutaneous coronary intervention (PCI), and is associated with a greater risk of complications (2, 3). Plaque modification procedures, such as rotational atherectomy (RA) and orbital atherectomy, improve vessel compliance and enhance stent optimization and expansion. Intravascular imaging demonstrates both plaque morphology and the outcome of stent optimization. The characteristics of coronary calcification based on intravascular imaging predict suboptimal stent expansion (4, 5) and determine the need for plaque modification. The role and benefit of routine use of intravascular imaging is not yet fully understood since the intravascular imaging catheter may not be able to pass through a cross severely calcified lesion. The cost of PCI is also significantly increased when adding both coronary imaging and RA. Conflicting results have been reported regarding the effect of the routine use of intravascular imaging guidance on major adverse cardiovascular events (MACE) compared to angiographic guidance. The results of some randomized trials in drug-eluting stent (DES) implantation reported that routine imaging guidance (intravascular ultrasound [IVUS], optical coherence tomography [OCT]) may not be recommended for all procedures (6, 7). Other studies reported that the use of intravascular imaging guidance facilitated larger postinterventional minimal luminal diameter and lower MACE, especially in complex lesion, such as chronic total occlusion, diffuse long lesion, bifurcation lesion and left main disease (8–20). Data supporting the benefit of intravascular imaging guidance compared to angiographic guidance in the patients with heavily calcified lesion undergoing RA-assisted DES implantation remain scarce. Accordingly, the aim of this study was to investigate the incidence of 1-year MACE compared between intravascular imaging guidance and angiographic guidance in patients undergoing RA-assisted DES implantation.

## Materials and Methods

### Study Design and Patients

This single-center retrospective cohort study enrolled consecutive patients who underwent RA with or without intracoronary imaging at the Faculty of Medicine Siriraj Hospital, Mahidol University, Bangkok, Thailand. The protocol for this study was approved by the Siriraj Institutional Review Board (SIRB) (COA number Si 349/2020). This study complied with all of the principles set forth in the Declaration of Helsinki (1964) and all of its later amendments. All consecutive patients aged >18 years who underwent rotational atherectomy (RA) and DES implantation during January 2016 to December 2018. Patients who underwent plain balloon angioplasty (POBA) or who received a bare metal stent were excluded. Operators who perform <100 PCI procedures per year were not permitted to participate in this study. Patients were divided in two groups based on RA with and without intracoronary imaging. The use of intracoronary imaging and the type of intracoronary imaging (IVUS or OCT) was decided based on operator discretion. In the angiographic guidance group, stent diameter and length were determined by visual estimation.

All patients received a bolus injection of heparin 100 unit/kilogram to maintain activated clotting time >250 s. Dual antiplatelet therapy with 81 mg/day aspirin with either 75 mg/day clopidogrel or 10 mg/day prasugrel or 90 mg twice a day ticagrelor was continued for at least a year after the procedure. The conventional 0.014-inch guidewire was replaced with a 0.009-inch ROTAWire^TM^ floppy guidewire (Boston Scientific, Marlborough, MA, USA) or a 0.009-inch ROTAWire^TM^ extra support guidewire (Boston Scientific). The initial burr size was chosen based on angiographically determined vessel size. A step-up approach starting with a 1.25, 1.5, and 1.75 burr was recommended according to European expert consensus on rotational atherectomy (21). In the intravascular imaging guidance group, burr size selection was based on preprocedural intravascular imaging if the lesion could be crossed with imaging. The selected burr speed was 140,000–180,000 revolutions per minute (RPM) with a run duration of 10–15 s. The final burr to artery ratio was targeted within 0.4–0.6. In cases where imaging could not cross the lesion before the procedure, preprocedural imaging was performed after RA. Non-compliance balloon or cutting balloon was routinely used for pre-dilatation before stent implantation. In the intravascular imaging guidance group, the stent diameter was determined by measuring the external elastic lamina diameter at the proximal and distal reference sites. In the angiographic guidance group, the stent diameter was determined by visual estimation. In the intravascular imaging guidance group, post procedure imaging was performed after stent implantation to evaluate for stent apposition, stent optimization, and procedural complication, such as dissection and tissue protrusion. A frequency-domain OCT system (ILUMIEN^TM^ OPTIS^TM^; Abbott Vascular, Santa Clara, CA, USA) was used for OCT-guided RA. For IVUS-guided RA, either an OptiCross^TM^ system (Boston Scientific) or an Eagle Eye Platinum^TM^ system (Philips Healthcare, Amsterdam, Netherlands) was used based upon operator discretion.

Baseline demographic and clinical characteristics were collected and recorded. Procedural characteristics, such as target vessel, stenosis diameter, type and size of stent, and RA details, such as number and burr size, and procedure duration were also recorded. We also collected procedural complications (angiographic dissection, perforation, or acute closure) and additional required interventions (prolonged balloon inflations, additional stent implantation, intra-aortic balloon pump [IABP], pericardiocentesis). Follow-up data were obtained from our center's database and from telephone calls made to patients or their family.

Quantitative coronary angiography (QCA) analysis was performed using a cardiovascular measurement system (QAngio XA 7.2; MEDIS, Leiden, Netherlands) by an experienced operator who was blinded to the patient's group. Optimal views of lesions were obtained at baseline and after the procedure at the same angle projection.

### Study Endpoints

We defined myocardial infarction (MI) according to the consensus definition of the Society of Cardiovascular Angiography and Interventions (22), and stent thrombosis according to the definite or probable criteria of the Academic Research Consortium (23).

The primary endpoint was 1-year MACE, which was defined as all-cause death, myocardial infarction (MI), stroke, or target vessel revascularization (TVR). The secondary endpoint was in-hospital MACE, which was defined as all-cause death, MI, stroke, or TVR before hospital discharge.

### Statistical Analysis

The sample size was calculated based on the following data from Gorol (24) and Sakakura (25): event rate of 15% in the angiographic guidance group (24), and an event rate of 1.33% in the intravascular imaging guidance group (25). Using this information, our power analysis and sample size calculation revealed that a sample size of 260 patients would yield 80% statistical power to detect the effect size between groups (alpha = 0.05, beta = 0.20).

Comparisons of continuous data with normal distribution were made using Student's *t*-test, and using Mann–Whitney *U* test for non-normally distributed data. Continuous variables are expressed as mean ± standard deviation (SD) or median and interquartile range (IQR). Categorical variables were compared using chi-square test or Fisher's exact test, and those results are presented as number and percentage. Outcomes are expressed as proportions, and their variability as 95% confidence intervals. Logistic regression analysis was used to determine the independent effect of variables on outcome. The following variables were included in the analysis: history of previous coronary artery bypass graft (CABG), stent length, maximum stent size, maximum balloon, left anterior descending artery lesion, number of RA used, maximum RA size, and imaging-guided RA DES placement. A *p* ≤ 0.05 was defined as indicating statistical significance. All statistical analyses were performed using SPSS statistics version 18 (SPSS, Inc., Chicago, IL, USA).

## Results

**Figure 1:**
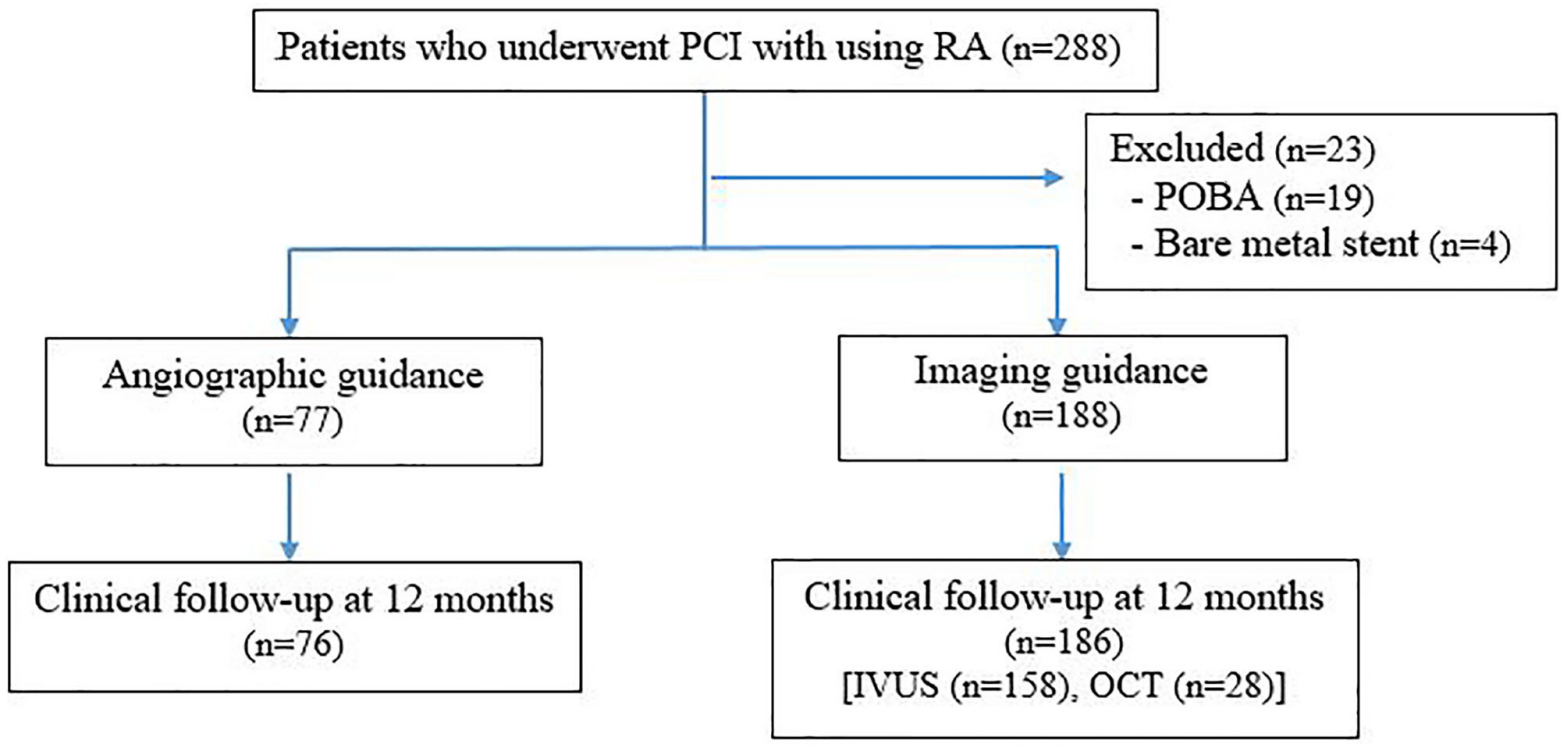
Flowchart of the patient enrollment process. PCI, percutaneous coronary intervention; RA, rotational atherectomy; POBA, percutaneous old balloon angioplasty; IVUS, intravascular ultrasound; OCT, optical coherence tomography.

Two-hundred and eighty-eight patients underwent RA-assisted PCI during the study period. Twenty-three patients were excluded because drug eluting stent was not deployed. Two hundred and sixty-five patients were enrolled, including 188 patients in the intravascular imaging guidance group, and 77 patients in the angiographic guidance group. A flow diagram showing the patient enrollment process is shown in Figure 1. One-year clinical follow-up was 98% in both groups, as follows: 186 of 188 patients in the intravascular imaging guidance group, and 76 of 77 patients in the angiographic guidance group.

Baseline demographic and clinical characteristics of patients are shown in Table 1. History of prior coronary artery bypass was significantly higher in the angiographic guidance group. Procedural characteristics are shown in Table 2. Reference vessel diameter, minimal lumen diameter and diameter stenosis by QCA before and after stenting were not significant between group. Patients in the intravascular imaging guidance group had a larger acute gain (1.91 vs 1.77, *p* < 0.039), larger stent diameter (3.00 *vs*. 2.75 mm, *p* < 0.001) and longer stent length (38.0 *vs*. 33.0 mm, *p* = 0.004) compared to patients in the angiographic guidance group. Patients in the intravascular imaging guidance group also had significantly more RA burrs (1.210 ± 0.42 *vs*. 1.070 ± 0.31, *p* = 0.005) and larger RA size compared to the angiographic guidance group. Radiation dose was lower in the intravascular imaging guidance group compared to the angiographic guidance group (2,727 *vs*. 3,335 mGy, *p* = 0.025). The success rates were similar between groups. Intravascular imaging findings are shown in Table 3.

**Table 1:** Baseline demographic and clinical characteristics compared between the angiographic and intravascular imaging guidance groups.

**Characteristics**	**Angiographic guidance**	**Imaging guidance (IVUS+OCT)**	***p***
	**(*n* = 77)**	**(*n* = 188)**	
Age (years)	74.48 ± 0.1	72.81 ± 0.2	0.169
Male sex	34 (44.2%)	104 (55.3%)	0.099
Diabetes mellitus	51 (66.2%)	109 (58.0%)	0.212
Hypertension	70 (90.9%)	180 (95.7%)	0.145
Dyslipidemia	53 (68.8%)	136 (72.3%)	0.566
Chronic kidney disease	11(14.3%)	39 (20.7%)	0.222
On renal replacement therapy	5 (6.5%)	11 (5.9%)	0.784
Previous PCI in target vessel	4 (5.2%)	16 (8.5%)	0.354
Previous CABG	12 (15.6%)	14 (7.4%)	***0.043***
Prior myocardial infarction	18 (23.4%)	49 (26.1%)	0.648
Smoker	16 (20.8%)	49 (26.1%)	0.364
Clinical syndrome			
STEMI	3 (3.9%)	3 (1.6%)	
Non-ST-ASC	26 (33.8%)	76 (40.4%)	0.333
Stable CAD	48 (62.3%)	109 (58.0%)	
Antiplatelet (P2Y12) or anticoagulant			
Clopidogrel	66 (85.7%)	159 (84.6%)	
Ticagrelor	5 (6.5%)	24 (12.8%)	0.106
Prasugrel	3 (3.9%)	2 (1.1%)	
Warfarin	3 (3.9%)	3 (1.6%)	

*Data presented as frequency and percentage or mean ± standard deviation*.

*A p < 0.05 indicates statistical significance (Bold)*.

*IVUS, intravascular ultrasound; OCT, optical coherence tomography; PCI, percutaneous coronary intervention; CABG, coronary bypass graft; STEMI, ST-segment elevation myocardial infarction; Non-ST-ASC, non-ST elevation acute coronary syndrome; CAD, coronary artery disease*.

**Table 2:** Procedural characteristics compared between the angiographic and intravascular imaging guidance groups.

**Characteristics**	**Angiographic guidance**	**Imaging guidance (IVUS+OCT)**	***p***
	**(*n* = 77)**	**(*n* = 188)**	
Target vessel			
LM	2 (2.6%)	6 (3.2%)	1.000
LAD	46 (59.7%)	140 (74.5%)	***0.017***
LCX	9 (11.7%)	18 (9.6%)	0.606
RCA	22 (28.6%)	39 (20.7%)	0.169
Diameter of stenosis (%)	83.31 ± 0.4	83.29 ± 0.7	0.991
CTO lesion	6 (7.8%)	12 (6.4%)	0.679
No. of burrs	1.070 ± 0.31	1.210 ± 0.42	***0.005***
Maximum burr size (mm)	1.50 (1.25-1.50)	1.50 (1.50-1.75)	***0.001***
QCA findings			
Before stenting			
Reference lumen diameter (mm)	2.610 ± 0.52	2.570 ± 0.61	0.603
Minimum lumen diameter (mm)	0.540 ± 0.34	0.510 ± 0.35	0.557
Diameter stenosis (%)	79.21 ± 2.4	80.41 ± 2.4	0.502
Lesion length (mm)	34.491 ± 7.35	37.291 ± 5.53	0.231
After stenting			
Reference lumen diameter (mm)	2.670 ± 0.48	2.740 ± 0.51	0.311
Minimum lumen diameter (mm)	2.320 ± 0.40	2.420 ± 0.44	0.104
Diameter stenosis (%)	12.68 ± 0.4	11.27 ± 0.9	0.218
Acute gain (mm)	1.770 ± 0.46	1.910 ± 0.46	***0.039***
Stent length (mm)	33.0 (22.0–49.0)	38.0 (30.0–53.7)	***0.004***
Maximum stent diameter (mm)	2.75 (2.50–3.50)	3.00 (3.00–3.50)	***<0.001***
Drug-eluting stent (DES) type			
Everolimus-eluting (Xience Prime, Xience ProX)	25 (32.5%)	50 (26.6%)	0.335
Zotarolimus-eluting (Resolute Integrity, Resolute Onyx)	18 (23.4%)	34 (18.1%)	0.325
Sirolimus-eluting (Orsiro, Firehawk, Ultimaster)	3 (3.9%)	41 (21.8%)	***<0.001***
Biolimus-eluting (BioMatrix Alpha, BioFreedom)	29 (37.7%)	52 (27.7%)	0.109
DES more than 1 type	2 (2.6%)	11 (5.9%)	0.358
Maximum balloon size (mm)	2.75 (2.50–3.00)	3.00 (2.75–3.50)	***<0.001***
Radiation dose (mGy)	3,335 (2,307–4,473)	2,727 (1,936–3,912)	***0.025***
Contrast volume (ml)	170 (140–200)	169 (130–217)	0.818
Procedural success (%)	76 (98.7%)	188 (100%)	0.291

*Data presented as frequency and percentage, mean ± standard deviation, or median (IQR)*.

*A p < 0.05 indicates statistical significance (Bold)*.

*IVUS, intravascular ultrasound; OCT, optical coherence tomography; LM, left main; LAD, left anterior descending; LCX, left circumflex; RCA, right coronary artery; CTO, chronic total occlusion; QCA, quantitative coronary angiography; DES, drug-eluting stent*.

**Table 3:** Intravascular imaging findings.

**Characteristics**	**Angiographic guidance**	**Imaging guidance (IVUS+OCT)**	***p***
	**(*n* = 77)**	**(*n* = 188)**	
Before stenting			
Proximal reference			
Minimum vessel diameter (mm)	NA	3.960 ± 0.69	NA
Maximum vessel diameter (mm)	NA	4.350 ± 0.74	NA
Distal reference			
Minimum vessel diameter (mm)	NA	3.170 ± 0.66	NA
Maximum vessel diameter (mm)	NA	3.520 ± 0.71	NA
Mean reference lumen CSA (mm^2^)	NA	6.422 ± 0.21	NA
Lesion minimum lumen diameter (mm)	NA	1.660 ± 0.43	NA
After stenting			
Minimum stent CSA (mm^2^)	NA	6.822 ± 0.72	NA
Malapposition (%)	NA	23 (16.8%)	NA
Stent edge dissection (%)	NA	10 (7.2%)	NA
Tissue protrusion (%)	NA	11 (8.0%)	NA

*Data presented as frequency and percentage, mean ± standard deviation*.

*IVUS, intravascular ultrasound; OCT, optical coherence tomography; CSA, cross-sectional area*.

### 1-Year Clinical Outcomes

**Figure 2:**
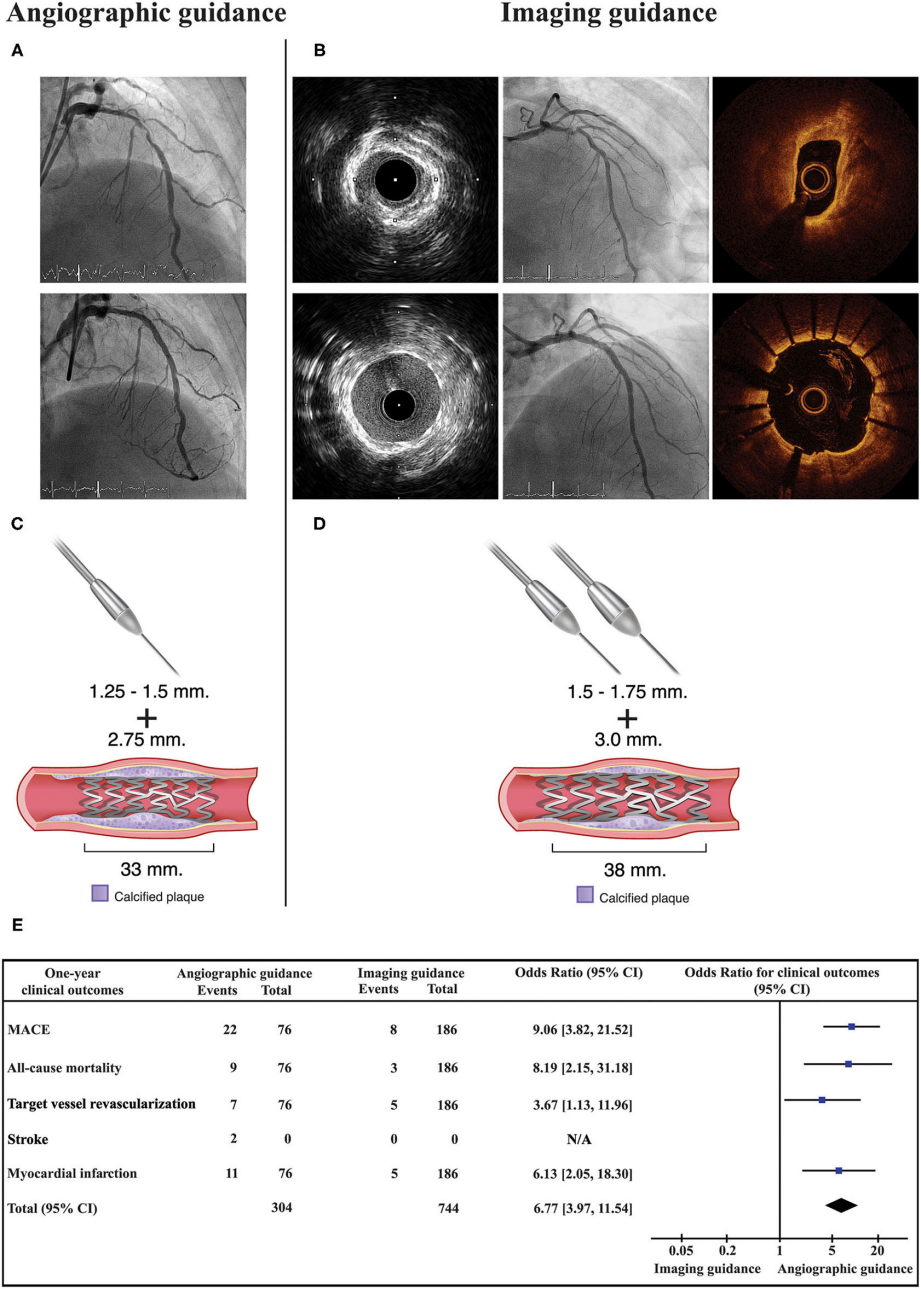
Central illustration. Compared strategy between the angiographic guidance and the intravascular imaging guidance in patients with heavy calcified lesion undergoing RA-assisted PCI. Angiographic guidance, coronary angiogram pre and post stent implantation is shown in **(A)**. Imaging guidance, coronary angiogram and intravascular imaging (IVUS or OCT) pre and post stent implantation is shown in **(B)**. **(C,D)** illustrated that the intravascular imaging guidance resulted in a greater number of RA burrs and larger RA burrs used, larger stent diameter and longer stent length. Forest plot presented odds ratio for one-year clinical outcomes compared between the angiographic guidance and the intravascular imaging guidance **(E)**. Intravascular imaging guidance had a significantly lower incidence of 1-year MACE. RA, rotational atherectomy; PCI, percutaneous coronary intervention; OCT, optical coherence tomography.

As shown in Figure 2 (Central illustration), the unadjusted 1-year rate of MACE was significantly lower in the intravascular imaging guidance group compared with the angiographic guidance group (4.3% [8 events] *vs*. 28.9% [22 events]; odds ratio (OR): 9.06, 95% confidence interval (CI): 3.82–21.52; *p* < 0.001). The 1-year unadjusted rates for all-cause death (OR: 8.19, 95% CI: 2.15–31.18; *p* = 0.002), MI (OR: 6.13, 95% CI: 2.05–18.30; *p* = 0.001), and TVR (OR: 3.67, 95% CI: 1.13–11.96; *p* = 0.031) were also significantly lower in the intravascular imaging guidance group than in the angiographic guidance group. The incidence of stroke was similar between groups. The overall 1-year rate of adjusted MACE was also significantly lower in the intravascular imaging guidance group compared with the angiographic guidance group (adjusted OR: 9.90, 95% CI: 3.78–25.93; *p* < 0.001). The observed significant decrease in MACE incidence was driven by reductions in all-cause death, MI, and TVR. Procedural complications, and active interventions after complications occurred were not significantly different between the angiographic and intravascular imaging guidance groups (Table 4).

**Table 4:** Unadjusted odds ratio of procedural complications and active interventions.

**Outcomes**	**Angiographic guidance**	**Imaging guidance (IVUS+OCT)**	**OR (95% CI)**	***p***
	**(*n* = 77)**	**(*n* = 188)**		
Procedural complications	6 (7.8%)	13 (6.9%)	1.14 (0.42–3.11)	0.802
Dissection at least type B	2 (2.6%)	7 (3.7%)	0.69 (0.14–3.39)	0.648
Perforation	3 (3.9%)	1 (0.5%)	7.58 (0.78–74.05)	0.082
Acute closure	0 (0%)	0 (0.0%)	NA	NA
Slow flow or no reflow	1 (1.3%)	5 (2.7%)	0.48 (0.06–4.19)	0.508
Active interventions after PCI complication	4 (5.2%)	13 (6.9%)	0.74 (0.23–2.34)	0.605
Prolonged balloon inflation	3 (3.9%)	2 (1.1%)	3.77 (0.62–23.02)	0.151
Additional stent required	3 (3.9%)	8 (4.3%)	0.91 (0.24–3.53)	0.894
Intra-aortic balloon pump	1 (1.3%)	4 (2.1%)	0.61 (0.07–5.50)	0.656

*Data presented as frequency and percentage*.

*A p < 0.05 indicates statistical significance*.

*IVUS, intravascular ultrasound; OCT, optical coherence tomography; OR, odds ratio; CI, confidence interval; PCI, percutaneous coronary intervention; NA, not applicable*.

## Discussion

The present study investigated the benefit of intravascular imaging guidance compared to angiographic guidance based on the incidence of 1-year MACE in patients with heavy calcified lesion who underwent RA-assisted DES implantation. The main findings of the study were, as follows: (1) Intravascular imaging guidance resulted in more appropriate final RA burr used according to target burr-to-artery ratio, with additional RA burrs used for adequate plaque modification; (2) Intravascular imaging guidance had larger acute gain, larger stent diameter and longer stent length; and, (3) Intravascular imaging guidance had a significantly lower incidence of 1-year MACE, driven by reduction of all-cause death, MI and TVR.

Intravascular imaging guidance facilitated an appropriate burr to artery ratio. An aggressive burr to artery ratio of >0.7 yields no advantage on clinical success or target vessel revascularization, but is associated with higher procedural complications (26, 27). European expert consensus (21) advice recommends a burr to artery ratio of 0.6. North American expert consensus (28) and Japanese expert consensus (29) recommend a burr to artery ratio of 0.4–0.6. Japanese expert consensus recommended the use of intravascular imaging if the operator aimed to achieve a burr to artery ratio of >0.6. Aggressive burr to artery ratio when the size of the vessel is overestimated can cause vessel perforation, dissection, and acute vessel closure. Angiographic guidance alone may underestimate true vessel size, especially in diffuse heavily calcified lesion. For pre-procedural assessment, intravascular imaging guidance helps in estimate true vessel size. Intravascular imaging provided not only proximal and distal reference vessel diameter, but also plaque characteristics, such as calcium distribution, thickness, and length. It is suggested that the operator opt for a larger RA burr without exceeding a burr to artery ratio of 0.6, and that the operator be more aggressive in the size of the device in more complex lesions. Even though a single burr strategy for lesion modification can be accomplished in the majority of cases. An additional RA burr will be required if calcified nodule, residual calcium thickness, and/or inadequate calcium fracture are detected by intravascular imaging. Intravascular imaging also gives an information of post-procedural assessment such as stent expansion, stent apposition, and complication detection. All these information from intravascular imaging guidance would lead to additional procedures such as post stent high pressure balloon dilatation in order to achieve adequate stent expansion and better stent apposition. This was supported by larger acute lumen gain with the minimum stent area (MSA) of 6.82 mm^2^ were achieved in the intravascular image guidance. Because of the complexity of the calcified lesions, stent malapposition still occurred in 16.8% of patients in the intravascular imaging guidance. The rate of stent malapposition could even higher in the angiographic guidance because of lacking information in preprocedural and post-procedural assessment from intravascular imaging. The observed benefit of intravascular imaging guidance PCI when using larger diameter stents and/or longer stents is similar to the result reported from the ADAPT DES trial (10), which compared between IVUS guidance PCI and angiographic guidance PCI in all-comers. It should be noted that radiation dose was significant lower in the intravascular imaging guidance.

Ali et al. (16) found a similar minimum stent area between the OCT guidance PCI and the IVUS guidance PCI. MACE was not significantly different between the intravascular imaging guidance PCI and the angiographic guidance PCI; however, that study recruited all-comers, and moderate to severe calcification was found in 16–26% of patients in each group. In the calcified lesions, calcium angle, calcium length and calcium thickness predicted stent expansion (5). IVUS has difficulty penetrating calcium depth but OCT has advantage in providing calcium thickness. Wang et al. (30) demonstrated that IVUS detected smooth surface with reverberation pattern was associated with the thinner calcium by OCT compared to IVUS-detected irregular surface without reverberation. Norihiro et al. (31) compared the OCT guidance PCI and the IVUS guidance PCI in patients with heavily calcified lesion who underwent RA. Percent stent expansion was larger in the OCT guidance RA group. They observed a non-significant trend toward lower TVR in the OCT guidance RA group. There were only 11 patients without intravascular imaging from 247 patients who underwent RA, and the clinical outcomes of the patients in the angiographic guidance PCI group were not provided. TVR was 6.8 and 11.6% in the OCT guidance and IVUS guidance PCI groups, respectively. In the present study, TVR was 2.7% in the intravascular imaging guidance PCI group. This observed difference in TVR between studies is likely due to differences in patients baseline characteristic. Twenty percent of patients were on hemodialysis in the Kobayashi study compared to 6% in our study.

The 1-year incidence of MACE in the intravascular imaging guidance group was significantly lower than in the angiographic guidance group. This was driven not only by TVR, but by all-cause death and MI. Shin et al. (32) conducted a meta-analysis of 2,345 patients with complex lesion who underwent DES implantation, and they found benefit of the IVUS guidance PCI over the angiographic guidance PCI for reducing MACE. They found a significant decrease in MI, but not in all-cause death. However, that meta-analysis included three randomized controlled trials that defined complex lesion as chronic total occlusion and long coronary lesion. Elgendy et al. (11) conducted a meta-analysis of seven randomized controlled trials that enrolled 3,192 patients. They also reported the benefit of the IVUS guidance PCI over the angiographic guidance PCI for reducing the incidence of MACE. In that study, TVR was significantly reduced, but only a non-significant trend was observed for mortality and stent thrombosis. Similar to the study by Shin, patients recruited in the Elgendy study was mainly chronic total occlusion and long lesion. Jang et al. (9) conducted a meta-analysis that included three randomized controlled trials and 12 observation studies with a total of 24,849 patients. Broader lesion characteristic, such as simple lesion, multivessel, chronic total occlusion, and left main (LM) disease, were included. In that study, the IVUS guidance PCI had lower MACE, all-cause mortality, MI, and TVR compared to the angiographic guidance PCI. That study reported no information regarding the percentage patients with heavily calcified lesion who underwent RA. Alsidawi et al. (33) conducted a meta-analysis of 10 randomized controlled trials that included 14,197 patients that underwent the intravascular imaging guidance PCI with drug eluting stent or bare metal stent placement. They found that the intravascular imaging guidance PCI significantly lowered the rates of death, MI, stent thrombosis, and MACE. However, the intravascular image guidance PCI had no effect on TVR in patients with DES, likely due to the effect of DES. The main findings of our study are consistent with those reported from previously published meta-analysis that found that the intravascular imaging guidance PCI reduced all-cause death, MI, TVR, and MACE. However, the results of our study provide additional benefit because we focused on patients with heavily calcified lesion who underwent RA-assisted DES placement. Superficial calcification is a predictor of worse prognosis. Fujino et al. (5) demonstrated OCT based calcium score identify lesion that need plaque modification. It is important process in decision making when dealing with heavy calcified lesion. Theoretically, either OCT or IVUS should be incorporated in a clinical algorithm to guide PCI strategy in calcified lesion to improve clinical outcomes. Intravascular imaging guidance PCI provides information specific to the necessity of plaque modification, true vessel sizing leads to use of appropriate burr-to-artery ratio, larger stent diameter and longer stent length, detection of procedural complications, and improving stent expansion and optimization.

### Study Limitations

This study has some mentionable limitations. First, our study was an observational, retrospective, single-center study, so our results should be interpreted with caution. The two study groups were non randomized, we cannot exclude the selection bias between patients with and without intravascular imaging guidance RA that could be influencing the study results. Second, pre-angiographic data based on QCA were similar between groups. Even investigators were blinded to patient's group. We could not exclude inter-operator variability in QCA measurement. Third, we included both IVUS guidance RA and OCT guidance RA so we could obtain as large a sample size as possible in the intravascular image guidance group. However, the number of patients was too small for subgroup analysis to detect significant difference between these two imaging techniques. Fourth, we do not have intravascular data in angiographic guidance patients to explain the cause of worse 1-year outcome compared to the intravascular imaging guidance group. There was no significant difference between groups for immediate procedural complications or in-hospital events. A possible cause of worse 1-year outcome could be stent malapposition and stent under expansion in angiographic guidance. Fifth, this study also lacked of a follow up imaging study that would explain the different in long-term procedural outcomes between groups. Further study in a randomized controlled trial is needed to confirm the benefit of intravascular imaging guidance in patients with heavily calcified lesion undergoing RA before recommended the routine use of intravascular imaging guidance in RA-assisted DES implantation.

## Conclusion

In patients with heavy calcified lesion undergoing RA-assisted DES implantation, intravascular imaging guidance significantly reduced the incidence of 1-year MACE, driven by reduction of all-cause death, MI, and TVR compared to angiographic guidance.

## Data Availability Statement

The raw data supporting the conclusions of this article will be made available by the authors, without undue reservation.

## Ethics Statement

The studies involving human participants were reviewed and approved by the Siriraj Institutional Review Board (SIRB) of the Faculty of Medicine Siriraj Hospital, Mahidol University. Written informed consent for participation was not required for this study in accordance with the national legislation and the institutional requirements.

## Author Contributions

NatW contributed to conception and design of the study and completed the writing review and editing. PB wrote the first draft of the manuscript. PB and KA collected the data. NatW, DT, CC, RPh, WT, NC, KT, AP, PaP, and PrP participated in the study. NatW, NamW, and KA contributed to the review of quantitative coronary angiography data and intravascular imaging data. NatW, PB, and RPo performed statistical data analysis and interpretation. All authors contributed to manuscript revision, read, and approved the submitted version.

## Conflict of Interest

The authors declare that the research was conducted in the absence of any commercial or financial relationships that could be construed as a potential conflict of interest.

## Publisher's Note

All claims expressed in this article are solely those of the authors and do not necessarily represent those of their affiliated organizations, or those of the publisher, the editors and the reviewers. Any product that may be evaluated in this article, or claim that may be made by its manufacturer, is not guaranteed or endorsed by the publisher.

## Abbreviations

CABG: coronary artery bypass graft
DES: drug-eluting stent
IVUS: intravascular ultrasound
MACE: major adverse cardiovascular event
OCT: optical coherence tomography
PCI: percutaneous coronary intervention
POBA: plain open balloon angioplasty
QCA: quantitative coronary angiography
RA: rotational atherectomy
TVR: target vessel revascularization.
